# Effects of Early Intervention on Visual Function in Preterm Infants: A Randomized Controlled Trial

**DOI:** 10.3389/fped.2020.00291

**Published:** 2020-06-04

**Authors:** Camilla Fontana, Agnese De Carli, Daniela Ricci, Francesca Dessimone, Sofia Passera, Nicola Pesenti, Matteo Bonzini, Laura Bassi, Letizia Squarcina, Claudia Cinnante, Fabio Mosca, Monica Fumagalli

**Affiliations:** ^1^Department of Clinical Sciences and Community Health, University of Milan, Milan, Italy; ^2^Fondazione IRCCS Ca' Granda Ospedale Maggiore Policlinico, NICU, Milan, Italy; ^3^Pediatric Neurology, Department of Human and Child Health and Public Health, Child Health Area, Catholic University UCSC, Rome, Italy; ^4^Department of Ophthalmology, National Centre of Services and Research for the Prevention of Blindness and Rehabilitation of the Visually Impaired, IAPB, Rome, Italy; ^5^Department of Statistics and Quantitative Methods, Division of Biostatistics, Epidemiology and Public Health, University of Milano-Bicocca, Milan, Italy; ^6^Fondazione IRCCS Ca' Granda, Ospedale Maggiore Policlinico, Occupational Health Unit, Milan, Italy; ^7^Department of Neurosciences and Mental Health, Fondazione IRCCS Ca' Granda Ospedale Maggiore Policlinico, Milan, Italy; ^8^Fondazione IRCCS Ca' Granda, Ospedale Maggiore Policlinico, Neuroradiology Unit, Milan, Italy

**Keywords:** preterm, early multisensory intervention, parental involvement, visual function, visual maturation

## Abstract

**Objectives:** To determine the effectiveness of an early intervention program in enhancing visual function in very preterm infants. Methods: We conducted a RCT. We included preterm infants born between 25^+0^ and 29^+6^ weeks of gestational age (GA), without severe morbidities, and their families. Infants were randomized to either receive Standard Care (SC) or Early Intervention (EI). SC, according to NICU protocols, included Kangaroo Mother Care and minimal handling. EI included, in addition to routine care, parental training according to the PremieStart program, and multisensory stimulation (infant massage and visual interaction) performed by parents. Visual function was assessed at term equivalent age (TEA) using a prevalidated battery evaluating ocular spontaneous motility, ability to fix and follow a target, reaction to color, stripes discrimination and visual attention at distance.

**Results:** Seventy preterm (EI *n* = 34, SC *n* = 36) infants were enrolled. Thirteen were excluded according to protocol. Fifty-seven infants (EI = 27, SC = 30) were assessed at TEA. The two groups were comparable for parental and infant characteristics. In total, 59% of infants in the EI group achieved the highest score in all the nine assessed items compared to 17% in the SC group (*p* = 0.001): all infants in both groups showed complete maturation in four items, but EI infants showed more mature findings in the other five items (ocular motility both spontaneous and with target, tracking arc, stripes discrimination and attention at distance).

**Conclusions:** Our results suggest that EI has a positive effect on visual function maturation in preterm infants at TEA.

**Trial Registration:** clinicalTrial.gov (NCT02983513).

## Introduction

Preterm infants, during NICU stay, face a stressful environment, as determined by intensive care, including excessive sensory stimulation and painful procedures (1–3), which may negatively impact early brain development (4), even in the absence of overt brain lesions, and may be implicated in impaired neurobehavioral outcomes (5, 6). Neuro-anatomical correlates, which are represented by micro-structural brain abnormalities, have been documented by advanced neuroimaging studies in preterms at term equivalent age (TEA) (7–10). These abnormalities are most likely related to the increased risk of neurodevelopmental, attentional or visuo-perceptual difficulties that preterm children can present at preschool and school age (11, 12).

Safeguarding brain development and maturation in preterms is therefore crucial for their neurodevelopment, and research has addressed new beneficial neuroprotective strategies.

Early intervention programs based on the concept of “individualized care” have effectively promoted brain maturation and neurodevelopmental outcomes (13, 14).

Based on the observation that early parenting is crucial in promoting early neurodevelopment, the parents' role in the NICU has been recently emphasized (15). However, the relationship between parents and their preterm infant during the neonatal period is “NICU mediated” (16), which can lead to a paucity of parent-infant interaction (16–18). In this framework, constructing a dyadic relationship is challenging (19) but potentially beneficial in reducing the effects of the NICU stressor environment both for the mother and the child (20–22).

Early interventions to improve mother-infant interaction seem to have the greatest potential to support child development; in this context different approaches have been proposed such as the PremieStart (23) that targets parental training to facilitate infant's well-being or the Family Nurture Intervention (FNI) that promotes mutual calm and emotional connection between mother and child (24).

No unique definition of early intervention exists in literature and this term has been widely used by several authors to refer to prevention-focused programs occurring in a period of high brain plasticity when interventions have the greatest influence on the child's neurodevelopmental outcome (25–27).

Among these interventions, multisensory stimulations have been recently suggested to enhance infants' neurodevelopment in different domains; in particular, infant massage, has been shown to accelerate the development of visual competences in preterms in the first year and to favor brain plasticity in infants at neurodevelopmental risk (28, 29).

The human visual system is a complex interaction between motor, perceptual and cognitive functions and visual development provide an early functional window into the development of infants' brain and connectivity (30–32).

Ricci et al. suggested that some features of visual function are more mature in preterm infants at TEA than they are in term-born infants suggesting that early experience has a role in the maturation of visual function and therefore supporting the possible beneficial role of early interventions in promoting it (33).

Other authors demonstrated the positive impact of infant massage on other aspects of neurodevelopment, including a reduction of stress behaviors (34, 35), even in those infants at a high neurological risk (36).

Only recently, the positive effect of an enriched environment on the brain and visual system development has been confirmed also by preclinical studies (37–39).

However, the effects of early intervention strategies, based on environmental enrichment that include parental involvement and positive multisensory stimulations, on visual function have not yet been investigated. Therefore, the primary outcome of the present study is to assess the effectiveness of an early intervention program in enhancing visual function in low-risk very preterm infants.

## Materials and Methods

We designed a randomized controlled trial (clinicalTrials.gov - Trial Registration Number: NCT02983513). The primary outcome of the study was to evaluate the effect of the Early Intervention program, that comprises enhanced mother-infant interactions combined with positive multisensory stimulations, on the visual function of preterm infants at term equivalent age (TEA), assessed with a specific neonatal visual examination. The secondary outcomes of the study included the evaluation of the effectiveness of the Early Intervention program on feeding behavior and long-term neurodevelopmental outcome.

The trial was approved by the Ethical Committee Milano Area B study on 14 March 2014. Written parental informed consent was provided for each infant in the study in accordance with the Declaration of Helsinki.

All preterm infants, consecutively born between 25^+0^ and 29^+6^ weeks of gestational age (GA) from April 2014 to January 2017 at the NICU, Fondazione IRCCS Ca' Granda Ospedale Maggiore Policlinico, Milano, were eligible for the study.

The exclusion criteria were: multiple pregnancy (triplets or higher); genetic syndromes and/or major congenital malformations; Necrotizing Enterocolitis (NEC) stage III (40); and major brain lesions, including Germinal Matrix Intraventricular Hemorrhage (GMH-IVH) >2° according to Papile (41) documented by early cranial ultrasound (cUS). The infants who, during their postnatal course, developed, retinopathy of Prematurity (ROP) > stage 2 (42) or extensive non-cystic white matter damage (NCWMD) at TEA brain MRI were excluded from analysis related to visual function.

Mothers were selected according to the following criteria: age over 18 years, good comprehension of Italian, no single-parent families, no obvious cognitive impairment or psychiatric disorders, and no drug addiction.

Infants were recruited after the first week of life and in a condition of clinical stability (i.e., no need for invasive mechanical ventilation and no active sepsis).

After obtaining parental written informed consent, infants were randomized to either receive Standard Care (SC) or an additional Early Intervention (EI). The randomization was performed using sealed envelopes that were prepared in groups of 10 through computer-generated randomization. The randomization sequence was concealed until the group allocation was assigned, and the examiner remained blinded for the entire study period.

SC, according to the NICU protocols, included Kangaroo Mother Care (KMC), nesting and minimal handling together with non-pharmacological pain management as required by the National Guidelines on pain control (43).

The EI program was delivered, in addition to routine care, during NICU stay by the same investigator (CF), and included parental training (based on the PremieStart program—that is focused on parental involvement to interpret infant's behavior and promote dyadic interactions) (23) together with enriched multisensory stimulation (infant massage and visual interaction) proposed by parents after a period of training. The parental training started 1 week after birth provided that the infant was clinically stable. After 3 weeks parents started infant massage, therefore promoting positive tactile stimulations, (twice a day until TEA) and from 34^+0^ weeks of GA onwards parents were trained to promote visual interaction (once a day until TEA). The present Early Intervention combines the effect of an empowered parental care with a positive and more active multisensory experience. A diary was given to parents to record intervention. A complete description of the EI protocol is available at (44).

During the study period, no specific interventions (e.g., Newborn Individualized Developmental Care Assessment Program—NIDCAP) to decrease stress were used.

The baseline characteristics, collected from hospital charts, included: gender, birth weight and GA, Small for Gestational Age (SGA) (45), twin birth, mode of delivery, Apgar score at 1 and 5 min, Clinical Risk Index for babies (CRIB) (46), number of days on invasive mechanical ventilation or on nasal continuous positive airway pressure (NCPAP) or High Flow nasal cannula, duration of hospital stay and GA at discharge.

The following neonatal morbidities were considered: ROP (42), NEC (40), Bronchopulmonary Displasia (BPD) (47), GMH-IVH (41) and sepsis (increased plasmatic levels of C reactive protein associated with a positive blood culture).

Family socioeconomic status (SES) was classified according to Hollingshead's criteria (48).

### Outcome Measure: Visual Assessment

At TEA [mean(SD): 40(3) weeks], infants underwent visual assessment according to the protocol developed by Ricci et al. (33) that evaluates: ocular movements both spontaneous and in reaction to a target, ability to fix and follow a target (horizontally, vertically and in an arc), ability to track a colored stimulus, stripes discrimination [evaluated using black and white stripes of increasing spatial frequency from 0.24 to 3.2 cycle/degree (49)] and visual attention at distance.

The best performance, according to the protocol, was defined as: mainly conjugated ocular motility, stable fixation, complete tracking, tracking of colored stimulus, discrimination of a spatial frequency over 2.4 cycles/degree and visual attention beyond 70 cm.

Infants were assessed in a single session (10 min) in a quiet environment with low light. The examination occurred when infants were in an alert behavioral state (50) and in a supine position. Responses for each of the nine items were recorded.

The examiner (ADC) was experienced in neonatal visual function assessment, using the proposed visual battery, and was blinded to the group assignment. No interactions occurred between parents and the examiner as he was not involved in infants' primary care and visual assessment was performed without parental participation.

### Statistical Analysis

Study's sample size was based on clinical feasibility and a power calculation: recruiting 70 infants would provide 80% power to detect a difference equal to 30% or more in visual performance between the groups (based on a 2-sided test with a = 0.05). We accounted for a 15% drop out.

Baseline characteristics were described as mean (standard deviation—SD), median and range, or number and percentage, as appropriate.

Visual function's comparison between the two groups was assessed for each item using Fisher's exact test.

Logistic regression models, used to estimate the relative risk of obtaining the best performance in each visual item, were run as sensitivity analysis, controlling for potential confounding effects. The results are presented as odds ratios (OR) and 95% CI.

All tests were two-tailed, and *p* < 0.05 was considered significant for all tests.

Statistical analyses were performed using R version 3.4.0 (R Foundation for Statistical Computing, Vienna, Austria).

## Results

**Figure 1:**
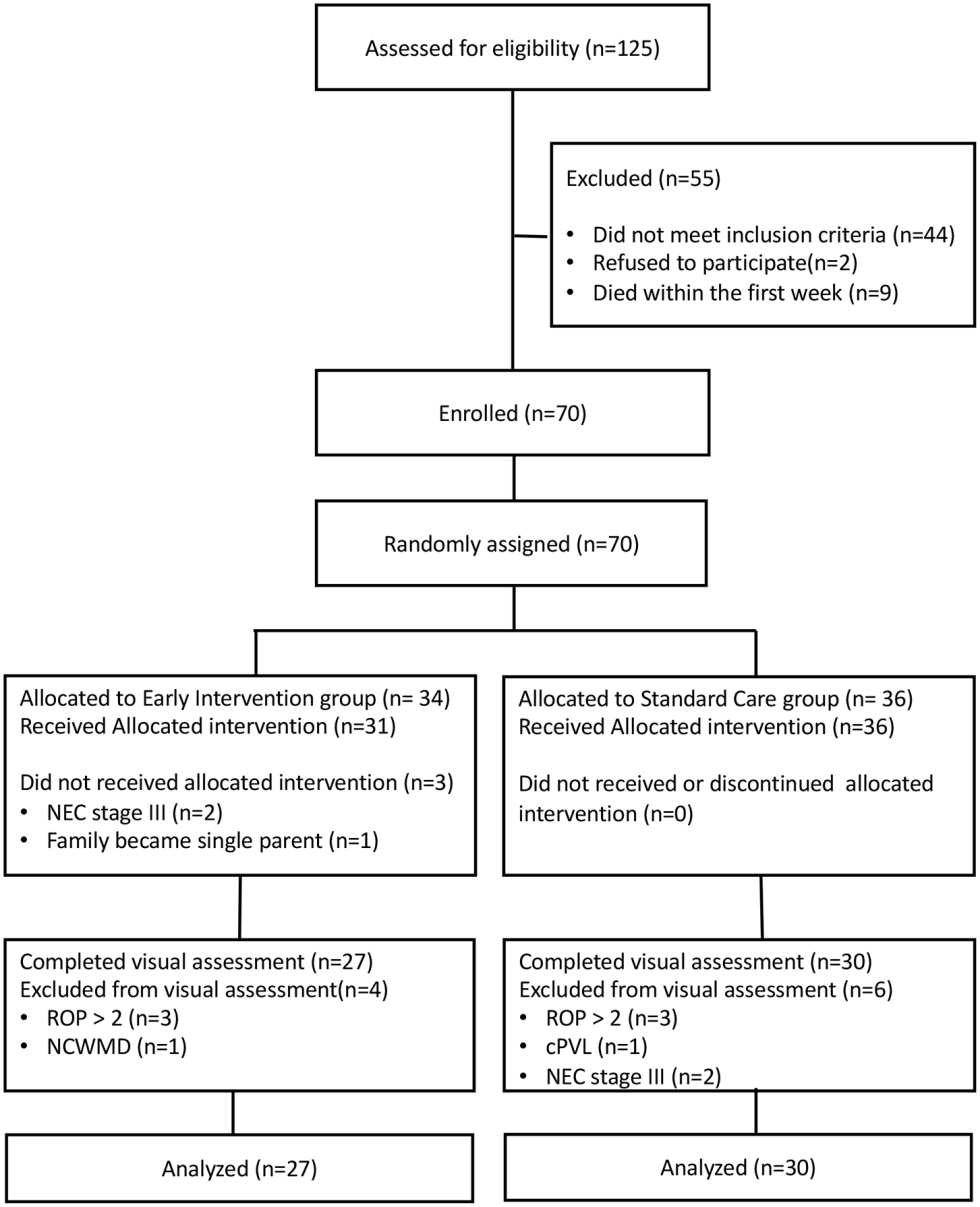
Flow chart of the study.

The flow chart of the study is reported in Figure 1. Overall, 70 infants (EI *n* = 34, SC *n* = 36) were recruited and randomized for intervention between April 2014 and January 2017. According to the protocol 3 infants allocated to EI did not receive treatment because: two developed NEC stage III and 1 family became single-parent after written informed consent was signed by both parents. All infants in the SC group received allocated treatment as part of routine clinical practice.

At TEA ten infants were excluded from visual assessment because: six developed ROP > stage 2 (three for each group), one in SC group developed cPVL, one in EI group presented NCWMD at MRI and two in SC group developed NEC stage III.

Fifty-seven infants (EI = 27, SC = 30) were assessed for visual functions at TEA.

Parent and infant characteristics were similar between the two groups (Table 1).

**Table 1:** Infants and maternal characteristics.

**Demographic feature**	**Early Intervention (*n* = 27)**	**Standard Care (*n* = 30)**
Gestational age at birth (weeks), mean (SD)	28.4 (0.9)	27.8 (1.3)
Birth Weight (g), mean (SD)	1,032 (249)	1,092 (312)
Male, *n* (%)	13 (48)	16 (53)
Singleton, *n* (%)	15 (56)	18 (60)
CRIB II score, mean (SD)	7.7 (1.7)	8.1 (2.3)
Apgar score at 1', median (range)	7 (4–9)	6 (2–8)
Apgar score at 5', median (range)	8 (7–10)	8 (5–9)
Cesarean Section, *n* (%)	25 (93)	26 (87)
Days of Mechanical Ventilation, mean (SD)	3.9 (7.5)	4.3 (6.3)
Days of NCPAP, mean (SD)	25.7 (13.7)	25.6 (14.0)
Days of High Flow Nasocannula, mean (SD)	15 (26.5)	7.2 (15.3)
Small for Gestational Age, *n* (%)	6 (22)	4 (13)
Sepsis, *n* (%)	11 (41)	11 (37)
Severe Bronchopulmonary Dysplasia, *n* (%)	8 (30)	5 (17)
GMH-IVH grade 1–2, *n* (%)	3 (11)	4 (13)
Retinopathy of prematurity <3, *n* (%)	1 (4)	6 (20)
Medical Necrotizing Enterocolitis, *n* (%)	0 (0)	1 (3)
Days of Hospitalization, mean (SD)	76 (24.0)	82.4 (35.1)
Length of stay in the incubator (days), mean (SD)	50.22 (15.5)	50.87 (19.9)
Gestational Age at Discharge, mean (SD)	39.2 (3.5)	39.6 (4.1)
Maternal Age, mean (SD)	33.9 (3.9)	33.8 (6.2)
SES, mean (SD)	50.7 (9.7)	44.8 (13.9)
Gestational Age at visual assessment, mean (SD)	40.7 (1.0)	41 (1.1)

In the EI group the massage therapy was started by parents at [mean (SD)] 32.1 (1.1) weeks of GA and carried out for 9.5 (2.1) times a week. Visual interaction was proposed starting from 34.9 (0.7) weeks of GA and performed 6.2 (1.6) times a week.

### Visual Function

The assessment was performed from June 2014 to April 2017 at TEA in the 2 groups [mean(SD) age: EI: 40.7 (0.99), SC: 41 (1.05)], and all infants completed the evaluation.

The infants in the EI group showed a more mature visual performance compared to the SC group.

In the EI group, 59% of the infants achieved the highest score possible on all 9 items of the assessment compared to 17% of the infants in the SC group (*p* = 0.001, Fisher Exact Test).

Main descriptive findings for each item of the assessment are presented below while the complete results are shown in Table 2.

**Table 2:** Visual assessment in the two groups.

**Neonatal Visual Assessment**	**Item Categories**	**Early Intervention (*n* = 27)**	**Standard Care (*n* = 30)**	***P*-value**
Spontaneous ocular motility	**Mainly conjugated**	**26 (96.3%)**	**21 (70%)**	0.013^◦^
	Occasional strabismus/occasional or lateral nystagmus	1 (3.7%)	9 (30%)	
	Intermittent strabismus/nystagmus	0 (0%)	0 (0%)	
	Continuous strabismus/nystagmus	0 (0%)	0 (0%)	
Ocular movements with target	**Mainly conjugated**	**23 (85.2%)**	**16 (53.3%)**	0.012^◦^
	Occasional strabismus/occasional or lateral nystagmus	4 (14.8%)	14 (46.7%)	
	Intermittent strabismus/nystagmus	0 (0%)	0 (0%)	
	Continuous strabismus/nystagmus	0 (0%)	0 (0%)	
Fixation	**Stable (>3 s)**	**27 (100%)**	**30 (100%)**	n.a.
	Unstable (<3 s)	0 (0%)	0 (0%)	
	Absent	0 (0%)	0 (0%)	
Tracking—Horizontal	**Complete**	**27 (100%)**	**30 (100%)**	n.a.
	Incomplete	0 (0%)	0 (0%)	
	Brief	0 (0%)	0 (0%)	
	Absent	0 (0%)	0 (0%)	
Tracking—Vertical	**Complete**	**27 (100%)**	**29 (96.7%)**	1^◦^
	Incomplete	0 (0%)	1 (3.33%)	
	Brief	0 (0%)	0 (0%)	
	Absent	0 (0%)	0 (0%)	
Tracking—Arc	**Complete**	**27 (100%)**	**24 (80%)**	0.025^◦^
	Incomplete	0 (0%)	6 (20%)	
	Brief	0 (0%)	0 (0%)	
	Absent	0 (0%)	0 (0%)	
Tracking colored stimulus	**Present**	**27% (100%)**	**30 (100%)**	n.a.
	Absent	0 (0%)	0 (0%)	
Stripes discrimination	**7–8 cards**	**21 (77.8%)**	**10 (33.3%)**	0.001^◦^
	5–6 cards	6 (22.2%)	15 (50%)	
	3–4 cards	0 (0%)	5 (16.7%)	
	<3 cards	0 (0%)	0 (0%)	
Attention at distance	**≥70 cm**	**20 (74.1%)**	**6 (20%)**	<0.001^◦^
	51–69 cm	6 (22.2%)	17 (56.7%)	
	30–50 cm	1 (3.7%)	7 (23.3%)	
	<30 cm	0 (0%)	0 (0%)	

“◦” *Fisher exact test*.

*The last column shows the p-value for a Fisher exact test comparing the best performance vs. all the others. The best performance is shown in bold*.

*Spontaneous ocular motility*: conjugated ocular motility was observed in 96.3% of infants in EI group and in 70% of infants in SC group.

*Ocular movements with target:* conjugated ocular motility was found in 85.2 and 53.3% of infants in EI and SC group, respectively.

*Fixation:* stable fixation was observed in all infants in both groups.

*Tracking:* horizontal tracking was complete in all infants in the two groups. The ability to track vertically was complete in all infants in EI group and in 96.7% of infants in SC group. Arc tracking was complete in the whole EI group and in 80% of infants in SC group.

*Reaction to a colored contrast target:* all infants in the two groups were able to track a colored target.

*Stripes discrimination:* the ability to discriminate cards 7–8 was observed in 77.8% of infants in EI group and in 33.3% of infants in SC group.

*Attention at distance:* the ability to keep attention on the target for more than 70 cm was observed in 74.1 and 20% of infants in EI and SC group, respectively.

To account for the possible uncontrolled effect of the distribution between EI and SC groups in terms of GA (*p* = 0.06) and ROP ≤ 2 (*p* = 0.06), considering that they might play a clinical meaningful role on visual development, adjusted logistic regression models were computed to compare infants that obtained the best performance in each item vs. all others. The multivariate analyses were computable for attention at a distance (OR, 14.9; 95% CI, 4.1–67.4; *p* < 0.001), stripes discrimination (OR, 7.5; 95% CI, 2.3–28.0; *p* = 0.001), ocular movements with target (OR, 5.9; 95% CI, 1.6–26.3; *p* = 0.01) and spontaneous ocular motility (OR, 13.7; 95% CI, 2.1–279; *p* = 0.02), and they confirmed the higher visual performance in EI group.

## Discussion

This is, to our knowledge, the first study focusing on the effects of a multisensory early intervention program on the maturation of visual function in preterm infants at TEA. Our findings suggest that early intervention strategies may have a positive effect on visual function and result in a possible acceleration of visual performance maturation.

More specifically, our data show that the difference between EI and SC group was obvious in some items but negligible in others. The discrepancy between the findings in the two groups of items can be easily explained by the known maturational pattern of individual function. Some items, such as fixation, horizontal and vertical tracking and tracking a colored stimulus, were already mature in the infants in our cohort, as expected at TEA and as observed in previous studies in low-risk preterms (33). Thus, all infants in the study achieved a maximum score, and no significant differences could be found between the groups.

In contrast, other items did not show a ceiling effect and could provide an opportunity to assess the differences in maturation in response to an intervention. In these items, whereas the SC group showed a level of maturation consistent with the previously reported range (33), the EI group showed higher scores, suggesting more mature findings.

Among the other items, some are dependent on subcortical structures, whereas others require cortical maturation; however, both showed acceleration in the EI group. More specifically, ocular motility and tracking for an arc at this age are mainly dependent on subcortical functioning. As these items are known to be influenced by experience (33, 51), the accelerated maturation of these abilities is likely to be partly related to the increased visual interactions that infants in the EI group experienced from 34 weeks postmenstrual age.

The combination of massage and increased visual stimulation may have influenced the maturation of more cortical aspects of visual function, such as stripes discrimination and attention at a distance, reported as being primarily dependent on postmenstrual age (32, 52, 53). Infants in the EI group, in fact, showed more mature responses in these items.

These findings are consistent with a previous study reporting the effect of infant massage on the maturation of visual function and brain electrical activity in low-risk preterms (54). In this study, infants received a multisensory intervention (body massage and auditory stimulation). Visual Evoked Potential (VEP) and Electroencephalogram (EEG) were performed before and after the massage, and functional visual assessment was performed at 3 months corrected age. The results showed that enriching the environment using a multisensory stimulation positively affects brain development and visual system maturation. Although the two protocols differ in the number and type of tactile stimulation, and in the actor performing the massage, our RCT confirms the potential benefit of a multisensory stimulation on the development of both cortical and subcortical visual function already at TEA.

The pathophysiological mechanisms underlying the observed effects of EI on visual function are still unclear, but hypotheses arise from previous studies highlighting how the environmental enrichment through positive sensory stimulation proposed during NICU stay, as infant massage or music listening, could promote infants' brain development and neurobehavior (27, 29, 54). Similar effects on microstructural and functional brain maturation could be hypothesized for the present EI program and future studies focused on brain development are advocated to better understand and interpret our findings.

This hypothesis is further endorsed by recent studies showing how interventions that support mother-infant relationship also benefit to infants' brain development at term age (55, 56). Mother and infant closeness is crucial for child development, and the Early Intervention here proposed may facilitate mother responsiveness to infant's needs and dyadic interactions through multisensory experiences. This hypothesis needs to be further confirmed but recently Ludwig and Welch (57) have identified in the emotional connection theory a possible construct underlying this close relationship and in the autonomic co-regulatory system a mechanism that explain the perinatal mother-infant emotional behavior. Early interventions that address dyadic interactions support this theory and have a demonstrated beneficial effect on preterm infants' neurodevelopmental outcome (58, 59).

To further confirm our short-term results, a longer follow-up is ongoing to determine if the observed differences in visual functions at TEA persist and whether they are associated with differences in neurodevelopmental outcomes.

Our study has some limitations. Based on previous evidence, we designed our RCT to study the effect of a combined multisensory approach (including both tactile and visual stimulation) to promote early visual function and child neurodevelopment; however, this has limited our ability to disentangle the contribution of each intervention as both have been proven to promote visual maturation. Second, due to the early nature of EI, a baseline assessment of visual function could not be performed; however, the randomization supports the homogeneity of the groups before intervention.

Another potential shortcoming of the study is the higher, but not significant, rate of ROP ≤ 2 observed in the SC group. However, this finding is unlikely to affect the robustness of our results, as demonstrated by the logistic regression models. Moreover, several studies reported that lower grades of ROP do not affect visual function (60, 61).

One of the strengths of our study is that we included only preterms with normal or mildly abnormal findings at cranial ultrasound, thereby excluding those with brain lesions who are more likely to develop visual disorders. This allowed us to avoid confounding factors (severe brain lesions and severe neonatal comorbidities potentially affecting neurodevelopment) when assessing the effects of EI on preterms, while previous studies evaluating the impact of PremieStart on neurodevelopment also included preterms with major brain lesion (13). On the other hand, these strict exclusion criteria led to a relatively small sample, which represents a limitation of the study and makes our risk estimates unstable. Moreover, recent evidences have reported an improvement in visual outcomes after visual interventions performed in the first month of life in children at high risk for Cerebral Palsy, who, therefore, should be included in future studies (62).

The novelty of our protocol relies on the active engagement of parents as first actors in the EI protocol; starting from PremieStart, they were then involved in performing massage and visual interaction, thus potentially helping parents to build a stronger dyadic relationship.

The EI here proposed, might therefore have ameliorated infants' ability to interact with their parents, also through improvement in visual function, with a positive effect on parents' responsiveness and promoting early bonding with their neonate.

Although not conclusive, we consider our results important to support a biologically well-described hypothesis; further confirmation is deserved from larger studies focused on possible effects of EI at both a biological and microstructural level and investigating long-term neurodevelopment.

## Conclusion

Our study, suggests that the positive effect of a multisensory approach can already be recorded at TEA for specific aspects of visual function, thus supporting the introduction of early intervention in the care of very preterm infants in addition to Standard Care.

## Data Availability Statement

The dataset generated for this study will be made available by the corresponding author on request in an anonymized version to guarantee low risk of patient identification.

## Ethics Statement

The study, involving human participant, was reviewed and approved by Ethical Committee Milano Area B. Written informed consent to participate in this study was signed by both parents.

## Author Contributions

CF and MF conceptualized and designed the study and drafted the initial manuscript. CF performed the early intervention. MF coordinated and supervised data collection. AD performed the visual assessment and collected data. DR designed the data collection instrument, helped in interpretation of results, and wrote the manuscript. FD and SP coordinated data collection. LB, LS, and CC helped in interpretation of results and wrote the manuscript. NP and MB carried out the statistical analyses. FM critically reviewed the manuscript for important intellectual content. All authors reviewed and revised the manuscript.

## Conflict of Interest

The authors declare that the research was conducted in the absence of any commercial or financial relationships that could be construed as a potential conflict of interest.
